# Extrinsic compression of the right external iliac artery secondary to iliac vein stenting

**DOI:** 10.1093/jscr/rjab193

**Published:** 2021-05-17

**Authors:** Syva Imayan Subramaniam, Adrian T Fung, David C Taylor

**Affiliations:** MD Undergraduate Program, The University of British Columbia, Vancouver, BC V6T 1Z3, Canada; Division of Vascular Surgery, Department of Surgery, University of British Columbia, Vancouver, BC V5Z 1M9, Canada; Division of Vascular Surgery, Department of Surgery, University of British Columbia, Vancouver, BC V5Z 1M9, Canada

## Abstract

Symptomatic extrinsic compression of the external iliac artery (EIA) is a rare complication of surgical intervention. Previous cases of EIA compression have presented in the postoperative period after orthopedic surgery or vascular stenting. We report a case of right EIA compression postvenous stenting causing acute limb ischemia.

## INTRODUCTION

Symptomatic extrinsic compression of the external iliac artery (EIA) has rarely been documented [1, 2]. The rarity of extrinsic compression is related to the robust structure of arterial walls compared with their venous counterparts; this is reflected in the extensive literature on venous compression compared with arterial compression [2–5]. Patients with EIA compression can present with claudication to acute limb ischemia [1, 2, 6–8]. Despite the rarity of iatrogenic EIA compression, it is important to maintain a high index of suspicion when patients present with acute ischemia after intervention near the iliac arterial system.

## CASE PRESENTATION

In this report we present a case of right EIA compression postvenous stenting. The patient is a 66-year-old man with a history of type 2 diabetes mellitus, hypertension, dyslipidemia, coronary arterial disease (status postcoronary artery bypass), chronic deep vein thrombosis (DVT), a previous history of bladder cancer treated with cystectomy and pelvic lymph node dissection. We obtained consent from the patient to publish his case details.

The patient was referred to the vascular surgery service for acute on chronic swelling of his right leg with bluish discoloration. He was recently assessed for progression of chronic DVT symptoms and had restarted oral anticoagulation. He underwent duplex ultrasound (DUS) examination, which confirmed complete thrombosis of the right iliofemoral and saphenous venous system. Due to his worsening symptoms and history of cancer, he was treated with injectable low molecular weight heparin, compression stockings and leg elevation for 2 months.

The patient remained severely symptomatic with significantly affected quality of life. There was no change of his disease on DUS. Magnetic resonance venogram revealed stenosis in the right common iliac vein as well as occlusion of the distal inferior vena cava (IVC). He was also noted to have a right-sided pelvic mass of uncertain etiology and oncology was involved. Given the significantly affected quality of life, the patient pushed for intervention, and an attempt at right iliofemoral venous angioplasty and stenting was arranged.

The patient underwent his right iliofemoral venous angioplasty and stenting under general anesthetic. Access through the femoral veins bilaterally was obtained with ultrasound. The right iliac venous was crossed with significant difficulty, as anticipated given the chronicity of the DVT. Upon crossing the lesion with up and over technique, we performed intravascular ultrasound to confirm the location of the occlusions. We then dilated the distal IVC to 16 mm, and a 16-mm Zilver Vena stent (Cook Medical, Bloomington, IN) was placed from below the left renal vein to above the iliac bifurcation. The right common and proximal external iliac veins were dilated to 14 mm, and the remainder of the external was dilated to 12 mm. A 10-mm Viabahn (Gore Medical; Newark, DE) stent was deployed from the distal external to the femoral vein. A 14-mm Wallstent (Boston Scientific; Natick, MA) was placed to cover the remainder of the proximal right common iliac vein.

**Figure 1:**
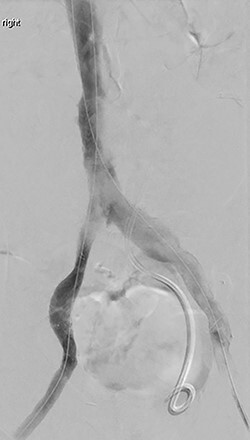
Completion venogram after recanalization and stenting.

A repeat intravascular ultrasound showed bilateral iliac vein stenosis at the level of the IVC bifurcation. Thus, we deployed 14-mm Wallstents in both iliac veins into the distal IVC. A complete venogram showed adequate flow through the external and common iliac systems bilaterally (Fig. 1). The total operating time was 6 h.

**Figure 2:**
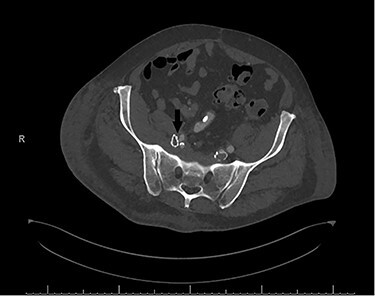
Postoperative computerized tomography angiogram with an arrow demonstrating likely point of compression of left EIA by venous stent.

In the immediate postoperative period, the patient was found to have weakness of the right foot, which was cool and pulseless. A computerized tomography angiogram showed well-positioned venous stents, but the right EIA was occluded from its origin to 4–5 cm distally (Fig. 2).

**Figure 3:**
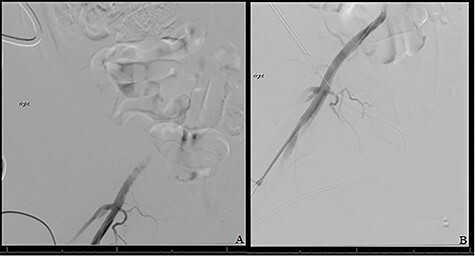
(**A**) Shows initial retrograde angiogram of right common femoral artery with occlusion at the level of the EIA. (**B**) demonstrates restoration of flow postangioplasty and stenting.

The patient was taken to the operating room immediately for percutaneous balloon angioplasty and stenting of the right EIA. The right femoral artery was accessed, and an angiogram confirmed occlusion of the right EIA. The occlusion was crossed, and an 8-mm Viabahn stent was placed to treat the occlusion. The completion angiogram showed complete revascularization of the right leg (Fig. 3). After hemostasis, the compartmental pressures of the right leg were noted to be normal, and normal Doppler signals were observed.

The patient experienced significant relief of leg swelling in the immediate postoperative period; however, at 1-month follow-up, there was recurrence of symptoms, and he was managed conservatively with anticoagulation and compression stockings.

## DISCUSSION

Although extrinsic EIA compression is a rare iatrogenic complication due to the rigid anatomy of the arterial wall, there have been reports of mass effect from abdominal masses [3, 6]. Compression of the EIA has been reported in one case caused by surgical retraction in an orthopedics case report [2] and two cases following venous stenting [1, 8]. The mechanism of compression of the EIA in the orthopedics case was related to the metal retractor compression of the external iliac against the psoas muscle.

In this case, the patient had a history of multiple pelvic surgeries, which could potentially increase regional scar tissue. This could have increased the rigidity of the soft tissue surrounding the EIA. The patient was also incidentally found to have a right pelvic tumor at the time of venous stenting workup, which would further increase the rigidity surrounding the right EIA. This risk profile combined with stenting of the right iliac venous system likely occluded the right EIA.

The decision to proceed with surgery took into consideration of the failure of conservative management resulting in continued poor quality of life. Although some would argue against treating the lesion, the patient did report debilitating venous symptomatology. Moreover, in the event of venous stent thrombosis, the thought was that the patient would return to baseline. However, the arterial complication does call into question the risk–benefit ratio of venous stenting in this context.

The decision to proceed with venous stenting should also consider a potential risk of EIA compression in patients with previous pelvic surgeries or existing pelvic masses. When anatomical risk factors are identified prior to venous stenting, it might be prudent to reconsider intervention based on the risk and benefit ratio. If intervention were pursued, the provider should maintain a high index of suspicion. In addition, close monitoring of the arterial status of the limb can help identify complications early and avoid the risk of limb ischemia and amputation.

## CONFLICT OF INTEREST STATEMENT

None declared.
